# The Report of the Parliamentary Committee on Poisons

**Published:** 1903-04-11

**Authors:** 


### THE REPORT OF THE PARLIAMENTARY COMMITTEE ON POISONS.

The report lately issued by the Departmental Committee appointed by the Lord President of the Council, to consider the present regulations concerning the sale of poisons, and to suggest any alterations they may deem expedient therein, must be described as a most disappointing document.

The main conclusions the Committee appear to have arrived at, after hearing the evidence and examining 26 witnesses are, that farmers and gardeners suffer inconvenience through the present restrictions on the sale of poisons, and that the conveyance of arsenic is lax. They therefore recommend that a new class of licensed but unqualified persons should be allowed to sell the poisons used in connection with agriculture, horticulture, or sanitation. At present, the sale of these poisonous preparations is restricted to duly qualified and registered chemists.

It must be conceded that the use of sheep-dips, weed-killers, and other poisonous preparations employed by agriculturalists has largely increased during the past twenty years, but to make this fact an excuse to relax the present restrictions on the sale of poisons, would not only be a most unwise and retrograde step, but also be dangerous to the public safety.

We quite agree with the statement made by a member of the Committee in his minority report, that "a review of the evidence is not convincing that under the present conditions any serious practical inconvenience has been experienced by the *bona fide* user in obtaining supplies of such poisonous compounds."

The Committee further recommend several very necessary additions to the present poison schedule, including cocaine, antifebrin and sulphonal, a list which might be further extended with advantage. The placing of cocaine and sulphonal under the poison restrictions, cannot fail to mitigate the terrible abuse of these drugs which has so largely increased in recent years; while the inclusion of antifebrin among poisons will be of value in putting a stop to the unrestricted sale of this potent remedy, which is at present so freely placed in the hands of the public in the form of "headache powders," that have already been responsible for several deaths.

The extraordinary suggestion made in the supplementary report by another member of the Committee, that the local authorities should grant licenses to suitable unqualified persons, to assist medical men in dispensing prescriptions, when it becomes necessary to do so in the absence of the doctor, scarcely needs serious consideration.

We cannot conceive what good could accrue from legalising a class of unqualified traders in poisons, such as the village grocer, ironmonger, or general store dealer; on the contrary, such a course could only be fraught with serious danger to the community.

The main safety of the public undoubtedly lies in the entire restriction of the sale of all poisonous substances to duly qualified persons, and it is to be hoped that when the legislature comes to deal with any amendment of the Pharmacy Act this course will be strictly adhered to, and that the regulations for the sale of poisons will be increased in stringency instead of being relaxed.

